# Small Bowel Obstruction Secondary to Metastatic Urothelial Cell Carcinoma With Plasmacytoid Features: A Case Report

**DOI:** 10.7759/cureus.63207

**Published:** 2024-06-26

**Authors:** Rafael A Guzman, Maikol Gonzalez, Sara Bakroun, Zinab Bakroun, Feras Othman, Joshua A Simon, Aruna Dash

**Affiliations:** 1 Medicine, St. George's University School of Medicine, True Blue, GRD; 2 General Surgery, Delray Medical Center, Delray Beach, USA; 3 General Surgery, University of Miami, Miami, USA; 4 Pathology, Delray Medical Center, Delray Beach, USA

**Keywords:** transitional cell carcinoma, metastatic small bowel cancer, small bowel resection, small bowel malignancy, plasmacytoid variant, plasmacytoid variant urothelial carcinoma, metastatic urothelial carcinoma, small-bowel obstruction, urothelial cell carcinoma, urothelial malignancy

## Abstract

Urothelial cell carcinoma (UCC) is a type of malignant cancer that affects thousands of people worldwide, especially those who smoke and have certain occupational exposures. Plasmacytoid urothelial carcinoma (PUC) is a rare histological variant of UCC that can present aggressively and insidiously. Small bowel obstruction secondary to malignancy is a rare presentation of UCC because the small bowel is a rare site of metastasis. We showcase a patient who presented with small bowel obstruction secondary to high-grade metastatic UCC with plasmacytoid features, exhibiting minimal urologic symptoms and no apparent risk factors. This case highlights the importance of high clinical suspicion for patients with possible malignancies that present with limited or unusual symptomatology and no risk factors. Further research into PUC to understand its symptoms and metastatic pattern is warranted to advance current early diagnostic criteria and further improve patient outcomes.

## Introduction

​​Urothelial cell carcinoma (UCC) is a malignant neoplasm that occurs in urothelial cells, otherwise known as transitional cells [1]. This type of cancer can occur in the bladder, renal pelvis, ureters, and parts of the prostatic urethra in males and the mucous urethra adjacent to the bladder in females [1]. However, 90% of UCC cases arise from the bladder and primary involvement of any other sites is rare [1]. Plasmacytoid urothelial carcinoma (PUC) is a rare form of aggressive UCC described in the literature as discohesive, oval-round shaped, eccentric, and indistinct nuclei with eosinophilic cytoplasm [2-4]. Risk factors for UCC include smoking (associated with >50% of all cases), industrial products that contain aromatic amines or polycyclic aromatic hydrocarbons, such as aniline dyes, rubber, or plastics, and to a lesser extent, environmental factors and diet [1,2,5]. Epidemiologically, UCC predominantly affects male patients with a higher incidence compared to females [2]. The typical age range for UCC is 59-75 years, with the plasmacytoid variant particularly manifesting in the age group of 62-75 years [2]. Advanced UCC typically metastasizes to the lymph nodes, bones, lungs, and liver in most cases [6]. The prognosis for typical UCC is an overall five-year survival of 71% ± 3 and the prognosis for the plasmacytoid variant is an overall five-year survival of 35% ± 10 at diagnosis [2]. Standard treatment for UCC is platinum-based neoadjuvant chemotherapy, such as methotrexate, vinblastine, adriamycin, and cisplatin (MVAC) or gemcitabine and cisplatin (GC) [7,8]. Small bowel obstruction (SBO) secondary to metastatic malignancy is common in patients aged 50.1 to 62.8 years and is seen commonly in patients with primary lobular breast cancer, malignant melanoma, and lung cancer [9]. Approximately 10% of malignant melanoma, 5% of lung cancer, and 9% of breast cancer metastasize to the small bowel [10,11]. We present a case of rare plasmacytoid UCC that metastasized to the small bowel and presented with SBO.

## Case presentation

A 58-year-old male patient with a past medical history of right-sided hydronephrosis with right ureteral stent presented to our institution with recurrent SBOs associated with diffuse abdominal pain, nausea, vomiting, and constipation. On physical exam, he had diffuse abdominal pain on palpation and distention. Vital signs were within normal limits. Lab findings were significant for leukocytosis and hypokalemia (Table 1).

**Table 1:** Abnormal laboratory results.

Labs	Patient	Normal range
WBC	13.0 uL	4.5-11.0 uL
Potassium (K+)	3.0 mEq/L	3.5-5.2 mEq/L

**Figure 1:**
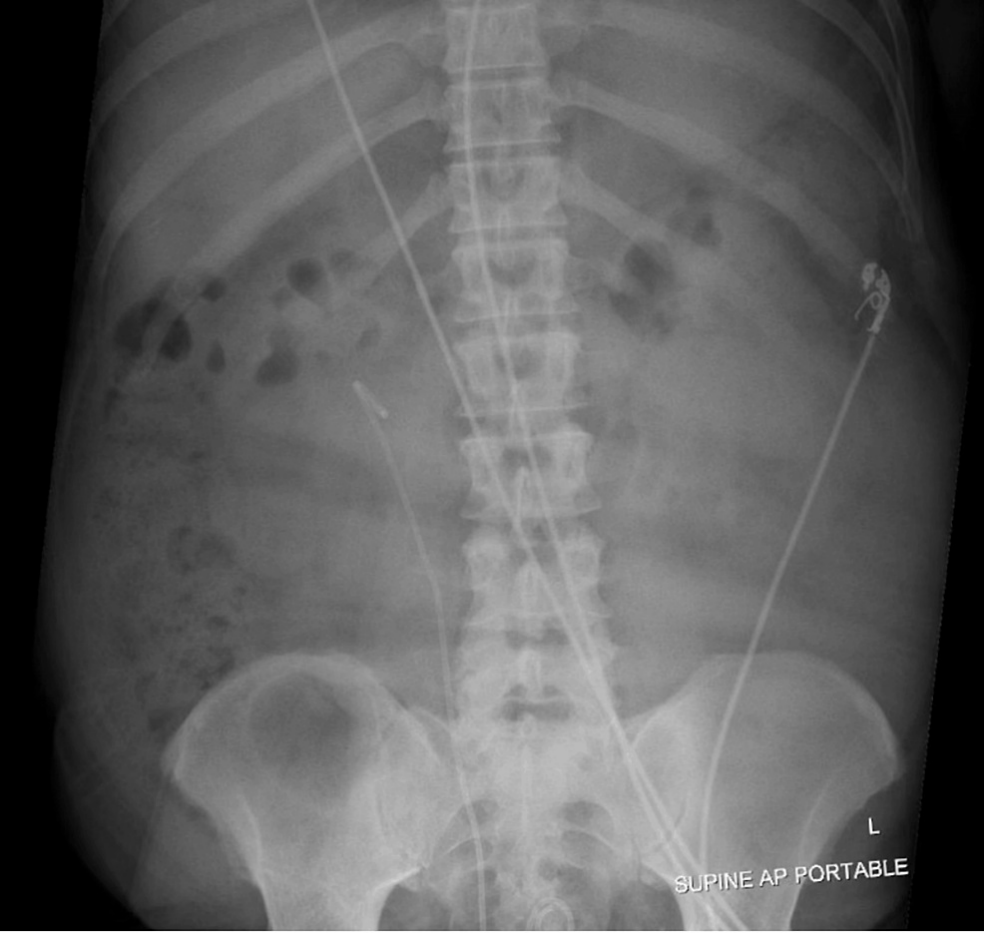
Abdominal X-ray showed a non-obstructive bowel gas pattern, moderate stable stool in the right colon, and right ureteral stent.

The abdominal X-ray was inconclusive (Figure 1).

**Figure 2:**
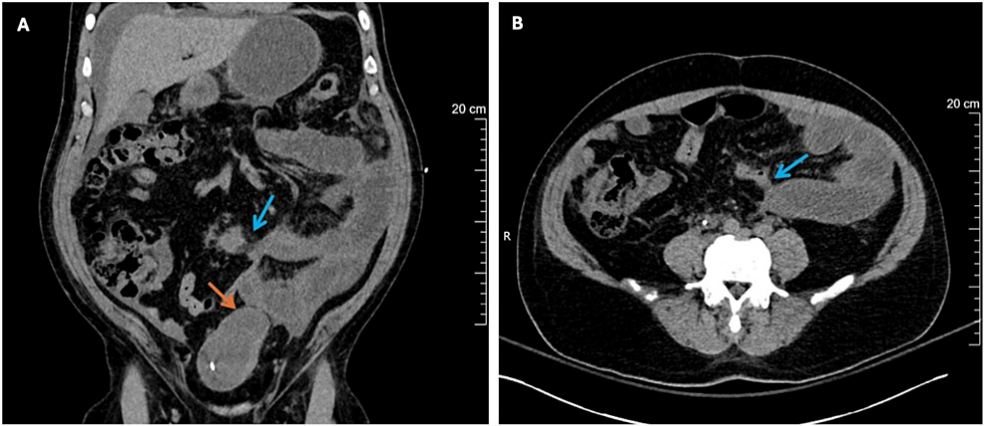
CT of the abdomen and pelvis. (A) Coronal view: The blue arrow points toward the transition point of the small bowel obstruction (SBO). The orange arrow points toward the left bladder wall thickening. (B) Axial view: The blue arrow points toward the transition point of the SBO.

Non-contrast CT of the abdomen and pelvis showed SBO with a left urinary wall mass concerning for malignancy (Figure 2).

**Figure 3:**
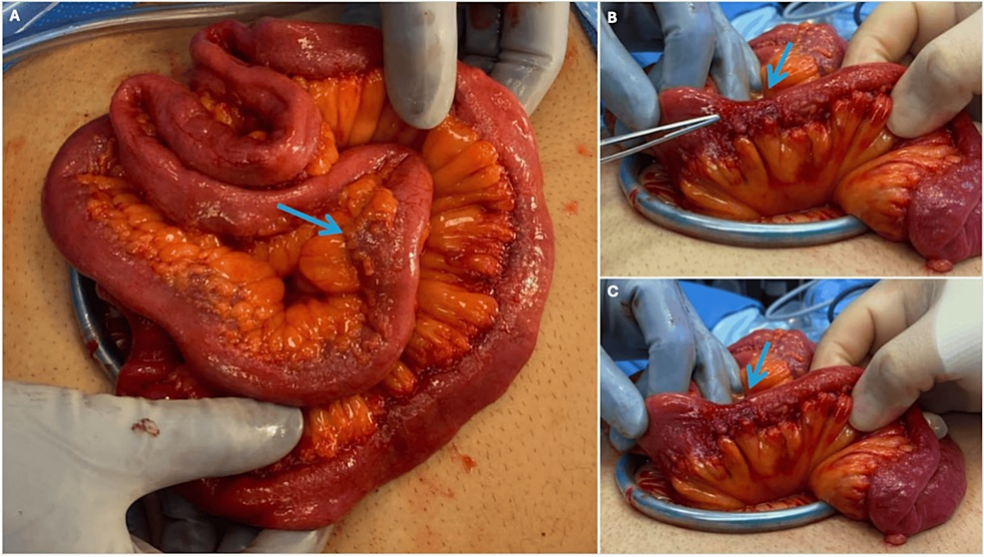
Small bowel mass as seen intraoperatively prior to surgical resection. (A) Top-down view: A blue arrow pointing toward the mass. (B & C) Side profile views: Blue arrows pointing toward the mass.

After five days of hospitalization, conservative management with nothing by mouth, bowel regimen, nasogastric tube, and intravenous fluids failed to improve the patient's SBO and we decided to do a diagnostic laparoscopy. During the procedure, a mass was found in the small bowel as the likely cause of the SBO. The operation was converted to open laparotomy and the mass was resected along with part of the small bowel, the specimen was sent for pathological analysis (Figure 3).

**Figure 4:**
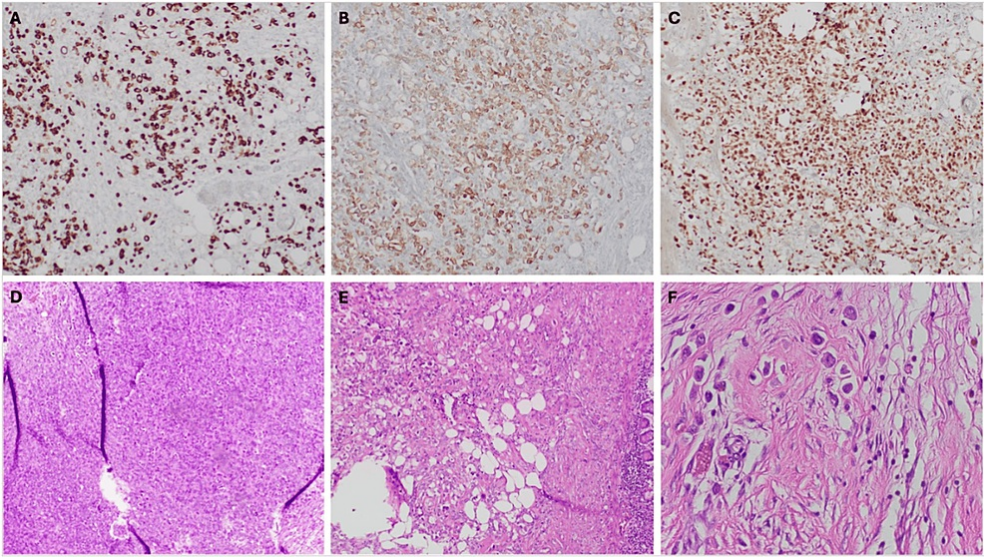
Histological slides obtained from surgically resected mass and margins. (A) Cytokeratin 7 (CK7)-stained cells. (B) Cytokeratin 20 (CK20)-stained cells. (C) GATA3-stained cells. (D & E) Hematoxylin and eosin (H&E)-stained cells (10x). (F) H&E-stained cells (40x) with appreciable plasmacytoid morphology.

Pathology reported poorly differentiated malignant neoplasm and infiltrates from serosa through submucosa into the mucosa, positive for CK7, CK20, and GATA3, consistent with metastatic high-grade UCC with plasmacytoid features. Both ends of surgical resection margins and radial/mesenteric margins were positive for poorly differentiated malignant neoplasm and subserosa and were suspicious for lymphovascular space invasion (Figure 4).

Intraperitoneal fluid culture collected during surgery was positive for *Candida albicans*, and he was treated with antifungal medication. The patient developed delirium during recovery and could not tolerate his nasogastric tube, which led to intractable nausea and vomiting. The combination of Candida peritonitis, total parenteral nutrition, and intractable nausea and vomiting led to acute kidney injury (AKI).

The case was presented at our institution's tumor board, and it was determined that the patient was a poor candidate for systemic therapy but could benefit from immunotherapy treatment. Treatment with pembrolizumab and enfortumab vedotin is recommended by the oncology team and the patient is being followed by the palliative care team to determine the best course.

## Discussion

In the US, 82,000 cases of bladder cancer are diagnosed yearly and it contributes to 16,700 deaths per year [12]. Of these cases, an estimated 90% are UCC and 1-3% of UCC cases are of the plasmacytoid variant [1,13]. PUC is diagnosed by observation of plasmacytoid cell morphology: discohesive, oval-round shaped, eccentric, and indistinct nuclei with eosinophilic cytoplasm [2-4]. Immunohistochemical staining of PUC is positive for CK7, CK20, CK138, GATA3, and loss of E-cadherin [13-15]. Histological analysis of this case showcased clear plasmacytoid morphology and positive staining for CK7, CK20, and GATA3, establishing the diagnosis of plasmacytoid UCC.

UCC typically presents with symptoms of dysuria, hematuria, and nocturia [16]. However, in many instances in the literature, PUC does not present with the characteristic findings of conventional UCC, lacking alert signs, such as gross hematuria, in some cases [17,18]. In this case, the patient presented with no gross urinary symptomatology. The only indication of bladder cancer that he did have was microscopic hematuria on urine analysis. Moreover, he presented with symptoms of SBO, which was ultimately found to be secondary to metastatic UCC with plasmacytoid features. SBO is a rare presentation of UCC because this cancer rarely metastasizes to the small intestines. A study looking at the metastatic pattern of bladder cancer showed that only 3% of primary bladder cancer metastasized to the intestines [6]. There are only a handful of reported cases of UCC presenting with SBO and only two cases (other than ours) of PUC presenting with SBO in the literature [19-22]. This makes SBO an extremely rare presentation of UCC, especially of the plasmacytoid variant, which is a small percentage of all UCC cases.

Both previously mentioned cases of SBO secondary to PUC had similar symptoms, including abdominal pain, vomiting, and nausea; neither presented with gross hematuria or other urologic symptoms, as in our case [20,22]. Curiously, other cases of PUC have presented with urinary symptoms like hematuria and dysuria [18,23]. Although there are very few cases of PUC in the literature, there does not seem to be a consistent symptom pattern in the cases that exist, and they can either have urological symptoms or have a silent presentation until the cancer metastasizes and starts to affect other organs.

Like our case, other similar cases of SBO secondary to metastatic PUC showed involvement of the retroperitoneal lymphovascular space, which became a highway for metastasis from the urothelium to the small bowel [20,22].

Major risk factors for UCC include smoking and exposure to aromatic amines and polycyclic aromatic hydrocarbons [1,2,6]. Our patient never smoked and was never exposed to products that contained these organic chemicals. However, he had undergone ureteral stenting for right-sided hydronephrosis prior to the known development of the bladder wall thickening. The previously mentioned case of SBO secondary to PUC had also undergone right ureteral stenting and subsequently developed PUC of the right ureter [20]. This raises the question of whether iatrogenic manipulation of the bladder and ureters increases the risk of developing PUC. Our patient also had a known history of prostate enlargement, which is known to cause bladder wall thickening through hypertrophy of the detrusor muscles of the bladder. The possible correlation between prostate enlargement and bladder cancer has yet to be explored in the literature [24].

Our patient is recovering from the abdominal surgery to remove the metastatic mass from his small bowel, proper staging of the cancer requires a full body PET scan, which could not be performed at the time, and the prognosis remains guarded for now. A study on the prognosis of 31 patients with PUC showed that cancer stages I-III had an overall survival of 45.8 months and cancer stage IV had an overall survival of 13.4 months [25]. In this study, four patients had metastatic cancer and all expired in six to 23 months [25].

The tumor board recommended the patient be started on immunotherapy with enfortumab vedotin plus pembrolizumab (Padcev and Keytruda) because the patient developed AKI during hospitalization likely due to reduced effective arterial volume in the setting of recently instituted total parenteral nutrition, Candida peritonitis, and intractable nausea and vomiting. Enfortumab vedotin plus pembrolizumab therapy has been proven effective at reducing tumor size and safe in patients who are ineligible for cisplatin-based therapy, such as patients with AKI [26].

This case report adds to the limited literature on plasmacytoid UCC metastatic to the small bowel. The case highlights how this condition can present with limited signs and symptoms of bladder cancer and might only manifest once the disease has advanced to later stages. It also showcases how PUC can present in patients with no obvious risk as this patient was not a smoker and had no known exposures that could cause bladder cancer. This combination of facts makes it difficult to diagnose this condition in a timely manner. It highlights the importance of having high clinical suspicion for rare diseases even if patients do not present conventionally. Further areas of study on this topic include the possibility of iatrogenic manipulation of the bladder causing bladder cancer and the possible association between prostate-related bladder cell hypertrophy and bladder cancer.

## Conclusions

UCC, also known as transitional cell carcinoma, is a type of malignant neoplasm that affects many patients yearly, especially those who smoke and have certain occupational exposures. Plasmacytoid UCC is a rare histological variant of UCC that presents aggressively and can be less predictable. Despite the conventional understanding of UCC, it is important to know that variations in presentation, such as SBO, can occur in the setting of UCC and even PUC. This highlights the importance of high clinical suspicion in cases where patients present with possible malignancy, even when they have no known risk factors or minimal or unusual symptomatology. Further research into this topic is warranted to understand the symptom and metastatic pattern of PUC, to advance possible early diagnostic modalities, and to further improve patient outcomes.
